# Diet-induced alteration of fatty acid synthase in prostate cancer progression

**DOI:** 10.1038/oncsis.2015.42

**Published:** 2016-02-15

**Authors:** M Huang, A Koizumi, S Narita, T Inoue, N Tsuchiya, H Nakanishi, K Numakura, H Tsuruta, M Saito, S Satoh, H Nanjo, T Sasaki, T Habuchi

**Affiliations:** 1 Department of Urology, Akita University Graduate School of Medicine, Akita, Japan; 2 AMED-CREST, Japan Agency for Medical Research and Development (AMED), Tokyo, Japan; 3 Research Center for Biosignal,Akita University Graduate School of Medicine, Akita, Japan; 4 Department of Clinical Pathology, Akita University Graduate School of Medicine, Akita, Japan

## Abstract

Fatty acid synthase (FASN) is a cytosolic metabolic enzyme that catalyzes *de novo* fatty acid synthesis. A high-fat diet (HFD) is attributed to prostate cancer (PCa) progression, but the role FASN on HFD-mediated PCa progression remains unclear. We investigated the role of FASN on PCa progression in LNCaP xenograft mice fed with HFD or low-fat diet (LFD), in PCa cells, and in clinical PCa. The HFD promoted tumour growth and FASN expression in the LNCaP xenograft mice. HFD resulted in AKT and extracellular signal-regulated kinase (ERK) activation and 5' adenosine monophosphate-activated protein kinase (AMPK) inactivation. Serum FASN levels were significantly lower in the HFD group (*P=*0.026) and correlated inversely with tumour volume (*P=*0.022). Extracellular FASN release was enhanced in the PCa cells with phosphatidylinositol 3-kinase (PI3K)/mitogen-activated protein kinase (MAPK) inhibition and AMPK signalling activation. FASN inhibition resulted in decrease of PCa cell proliferation through PI3K/MAPK downregulation and AMPK activation. Furthermore, AMPK activation was associated with FASN downregulation and PI3K/MAPK inactivation. Clinically, high FASN expression was significantly associated with high Gleason scores and advanced pathological T stage. Moreover, FASN expression was markedly decreased in the PCa response to androgen deprivation therapy and chemotherapy. HFD modulates FASN expression, which may be an important mechanism in HFD-associated PCa progression. Furthermore, a critical stimulatory loop exists between FASN and the PI3K/MAPK system, whereas AMPK signalling was associated with suppression. These may offer appropriate targets for chemoprevention and cancer therapy in HFD-induced PCa.

## Introduction

Prostate cancer (PCa) is the most commonly diagnosed cancer and the second most common cause of cancer-related death in the United States.^1^ Although the incidence of PCa is lower in Japan than in western countries, it is increasing.^2^ Many epidemiological studies have shown that a high-fat diet (HFD) or obesity is associated with PCa incidence and progression.^3, 4^ Furthermore, several studies have indicated that HFD intake may affect gene expression, cellular activity and the circulating levels of biological factors through the hyperactivation of the phosphatidylinositol 3-kinase (PI3K) and mitogen-activated protein kinase (MAPK) signalling pathways known to be involved in prostate carcinogenesis.^5, 6, 7, 8^

Fatty acid synthase (FASN) is a metabolic enzyme that catalyzes the *de novo* synthesis of fatty acids; it is regulated by sterol regulatory element-binding proteins (SREBPs).^9, 10, 11^ In addition, FASN has been shown to be upregulated by androgens and to promote LNCaP cell proliferation,^12, 13^ and FASN expression known to be regulated by PI3K and MAPK signalling pathways.^14, 15^ Furthermore, a recent study indicated that HFD-induced obesity increased melanoma progression through modulation of FASN expression,^16^ whereas dietary soy, vitamin D2 and green tea inhibited FASN expression in various types of cancer cells.^17, 18, 19^ Collectively, HFD and other dietary components may be associated with PCa progression through altered FASN expression. However, the precise underlying mechanism is poorly understood.

Human FASN is a 270 kDa cytosolic protein that is also found in the extracellular space.^20, 21^ For example, FASN has been found in both the culture supernatants of human breast cancer cells and the serum of breast cancer patients.^22, 23^ Extracellular FASN levels have been associated with FASN expression in the adipose tissue of patients with type 2 diabetes.^24^ In addition, 5' adenosine monophosphate-activated protein kinase (AMPK), which is key to maintaining intracellular energy balance,^25^ has been reported to have a substantial role in extracellular FASN release and cellular energy restoration in response to increased AMP:adenosine triphosphate ratios.^26^

In this study, we investigated FASN expression in LNCaP xenograft mice fed with HFD and low-fat diet (LFD). The results indicate that FASN expression is enhanced in xenograft tumour cells under HFD conditions, and that it is regulated by PI3K, MAPK and AMPK signalling pathways. Clinically, FASN expression was correlated with PCa progression. These results suggest that FASN and the related signalling pathways are involved in PCa progression, especially under HFD conditions.

## Results

### HFD increased tumour growth and expression of FASN and SREBP-1 as well as decreasing serum FASN levels in the LNCaP xenograft mice

**Figure 1:**
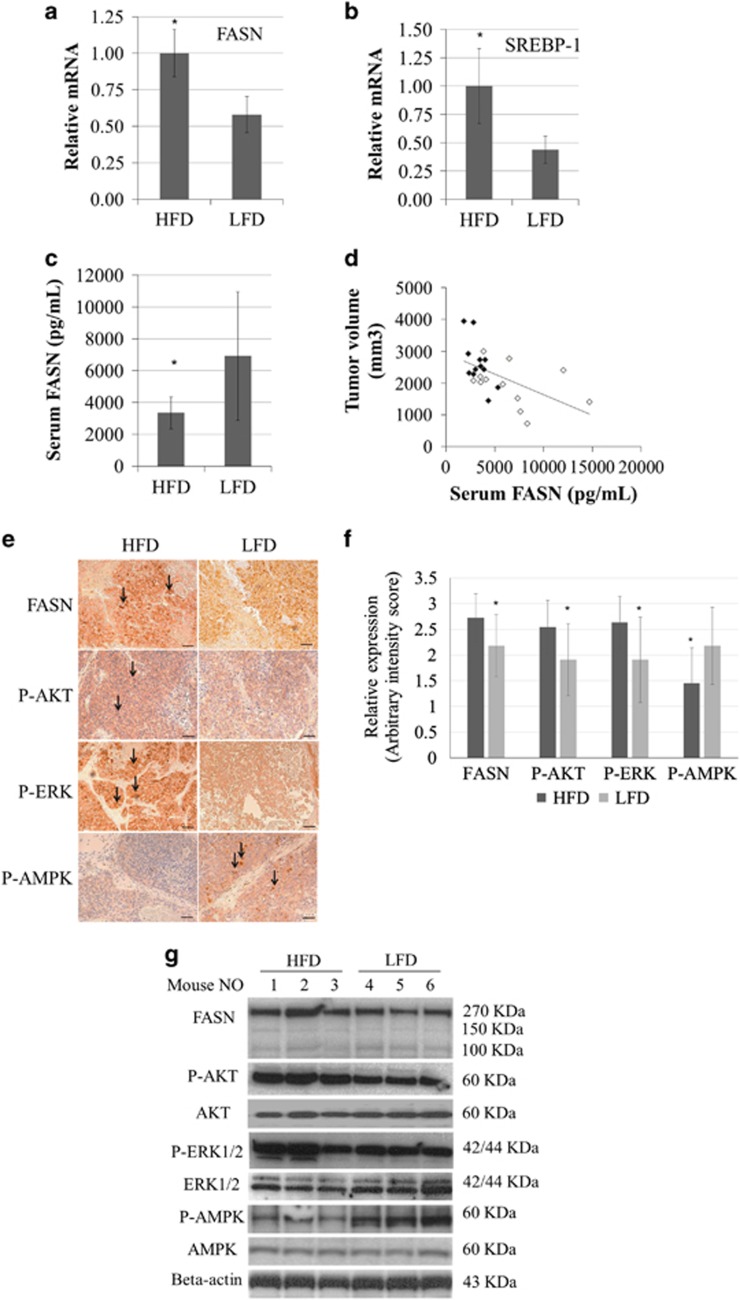
The expression of FASN and its related signal pathways in LNCaP xenograft mice under HFD or LFD conditions. LNCaP xenograft mice were generated, and those with palpable tumours were randomly assigned to HFD and LFD groups (12 mice per group). After the 14-week diet experiments, the tumour and serum samples were separated. (**a**, **b**) mRNA expression of *FASN* and *SREBP-1* in xenograft tumours was quantified by quantitative reverse transcription–PCR and the relative mRNA expression ratio was compared against beta-actin in the two diet groups. *P<0.05. (**c**) The serum FASN concentrations were measured by the human FASN ELISA kit. The mean serum FASN level was significantly lower in the HFD group than in the LFD group: 3344.6±1005.8 and 6666.4±3685.8 pg/ml, respectively (*P=*0.026). (**d**) Serum FASN levels were individually plotted for each animal against tumour volumes from xenograft mice and the Pearson correlation coefficient (*r*) was calculated using SPSS version 12 software (total, *r=*0.642, *P=*0.022; HFD group (closed circle), *r*=−0.618, *P=*0.024; LFD group (open circle), *r*=−0.439, *P=*0.154). (**e**) Xenograft tumour sections from mice in the HFD and LFD groups underwent immunohistological staining with anti-human FASN, P-AKT (Ser^473^), P-ERK (Thr202/Tyr204) and P-AMPK (Thr172) antibody (bar, 100 μm). The higher staining area is indicated (arrows). (**f**) The immunohistostaining intensity in the xenograft cancer cells was scored on a semiquantitative scale. The mean intensity score of FASN, P-AKT (Ser^473^) and P-ERK (Thr202/Tyr204) was significantly higher in the HFD group compared with the LFD group, but the mean intensity score of P-AMPK (Thr172) was markedly lower in the HFD group. (**g**) Equal amounts of proteins from the xenograft tumour were evaluated with western blot with anti-FASN, anti-AKT, anti-P-AKT, anti-ERK, anti-P-ERK, anti-AMPK, anti-P-AMPK and anti-beta-actin.

We generated an LNCaP xenograft mouse model by inoculating 1 × 10^6^ of LNCaP cells, and dividing the mice into HFD and LFD groups (Supplementary Table S1), with 12 mice per group. Although there was no significant difference in the level of food consumption in the two diet groups, the caloric consumption was significantly higher in the HFD group than the LFD group (10.4±0.4 and 9.3±0.5 kcal/day/mouse, respectively, *P=*0.034; Supplementary Table S2). At week 14 (end of the dietary experiments), tumour growth was significantly enhanced in the HFD group compared with the LFD group (*P=*0.025, Supplementary Figure S1), but there were no significant differences in serum levels of insulin and insulin-like growth factor 1 (IGF-1) in the two groups, consistent with the results of our previous study.^6^ Next, we investigated the expression of FASN and SREBP-1, the critical transcriptional factor for FASN, in the xenograft mice. *FASN* and *SREBP-1* mRNA expression was 1.8-fold and 2.1-fold higher in xenograft tumours of the HFD group compared with the LFD group, respectively (*n*=12 per group, *P*<0.05; Figures 1a and b). Similarly, the expression of FASN was higher in xenograft tumours of the HFD group than the LFD group (Figures 1e–g), whereas the mean serum FASN level was significantly lower in the HFD group than in the LFD group (3344.6±1005.8 and 6666.4±3685.8 pg/ml, respectively, *P=*0.026; Figure 1c). In addition, serum FASN levels were inversely correlated with tumour volumes (total, *r=*0.642, *P=*0.022; HFD group, *r*=−0.618, *P=*0.024; LFD group, *r*=−0.439, *P=*0.154; Figure 1d). These findings suggest that HFD enhances FASN expression in PCa cells and decreases the extracellular excretion of FASN while enhancing PCa progression.

### Upregulation of P-AKT and P-ERK as well as downregulation of P-AMPK in LNCaP xenograft tumours in mice under HFD conditions

Several studies have indicated that the dysregulated PI3K/AKT, extracellular signal-regulated kinase (ERK)/MAPK and AMPK signalling pathways are associated with PCa progression, and that HFD can activate AKT and ERK signalling.^27^ To explore the expression of protein kinase pathways in HFD-induced PCa tumour growth, we investigated the expression of AKT, P-AKT, ERK, P-ERK, AMPK, P-AMPK and Ki67 in xenograft tumours by immunohistochemistry and/or western blotting (Figures 1e–g; Supplementary Figures S2 and S3). P-AKT, P-ERK and the Ki67 positivity were significantly upregulated, whereas P-AMPK was significantly downregulated in the HFD group compared with the LFD group (Figures 1e–g, Supplementary Figure S3). In addition, there were no statistical differences in the expression of AKT, ERK and AMPK in the two diet groups (Supplementary Figure S2). These results suggest that HFD promoted PCa growth along with the modulation of signalling pathways, such as PI3K/AKT, ERK/MAPK and AMPK.

### Altered FASN expression through AKT and ERK inhibition as well as AMPK activation in PCa cells

**Figure 2:**
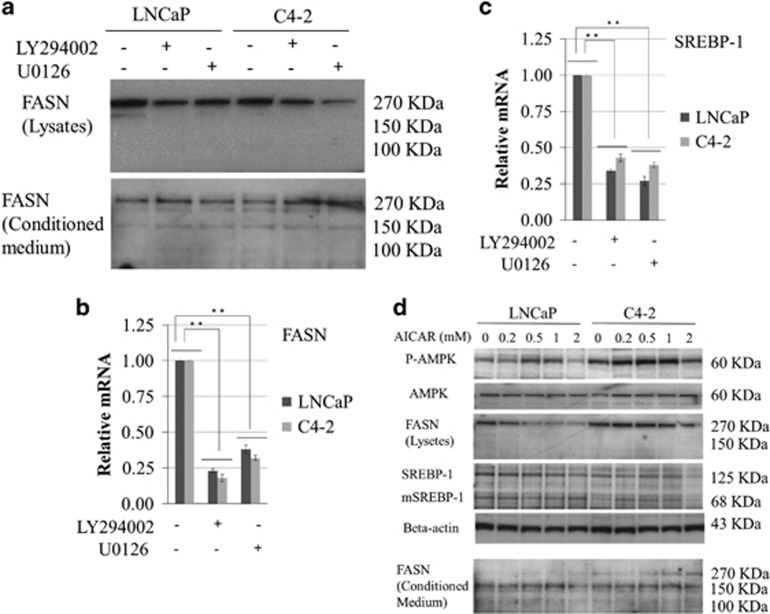
Effect of PI3K (LY294002) and MAPK (U0126) inhibitors and an AMPK activator (AICAR) on FASN expression in LNCaP and C4-2 cells. LNCaP and C4-2 cells were cultured in Dulbecco's modified Eagle's medium (DMEM) containing 10% fetal bovine serum (FBS) with and without 5 μM LY294002, 0.5 μM U0126 (**a**–**c**), or AICAR (**d**) at the indicated concentration for 24 h. (**a**) An equal amount of protein from the cells and conditioned medium was subjected to anti-human-FASN antibody. Total RNA was extracted from the cells and *FASN* (**b**) and *SREBP-1* (**c**) mRNA levels were measured by quantitative reverse transcription–PCR, and compared with those of untreated cells. **P<0.01. (**d**) The protein from the cultured cells and the supernatant was subjected to anti-AMPK, anti-P-AMPK, anti-FASN, anti-SREBP-1 and anti-beta-actin.

Next, we investigated the role of AKT and ERK signalling on FASN expression. Treatment with 5 μM of the PI3K inhibitor LY294002 or 0.5 μM of the MAPK inhibitor U0126 significantly decreased the intracellular FASN protein (as the 270 kDa intact form). However, FASN expression (as the 270 kDa intact and 100 and 150 kDa degraded forms) in the conditioned medium was increased in the LNCaP and C4-2 cells (Figure 2a). Furthermore, the expression of *FASN* and *SREBP-1* mRNAs was significantly lower in the LNCaP and C4-2 cells treated with LY294002 or U0126 compared with the parental cells (*P*<0.01; Figures 2b and c). 5-Amino-4-imidazole carboxamide riboside (AICAR), which is a pharmacological activator of AMPK, stimulated extracellular FASN release in breast cancer cells;^26^ therefore, we investigated the role of AMPK signalling in FASN and SREBP-1 expression in PCa cells treated with AICAR. Under these conditions, the protein expression of P-AMPK was increased in the LNCaP and C4-2 cells in a dose-dependent manner to an efficient maximum dose of 1 mM (Figure 2d). Furthermore, AICAR treatment resulted in expression of intracellular FASN (270 kDa intact form) and downregulation of the precursor (125 kDa) and mature (68 kDa) forms of SREBP-1, whereas FASN protein expression in the conditioned medium (mainly the 150 kDa degraded form) was increased in the LNCaP and C4-2 cells in a dose-dependent manner (Figure 2d). These findings suggest that the FASN expression was upregulated by increased PI3K/MAPK signalling or decreased AMPK signalling.

### FASN expression and crosstalk among PI3K, ERK and AMPK signalling

**Figure 3:**
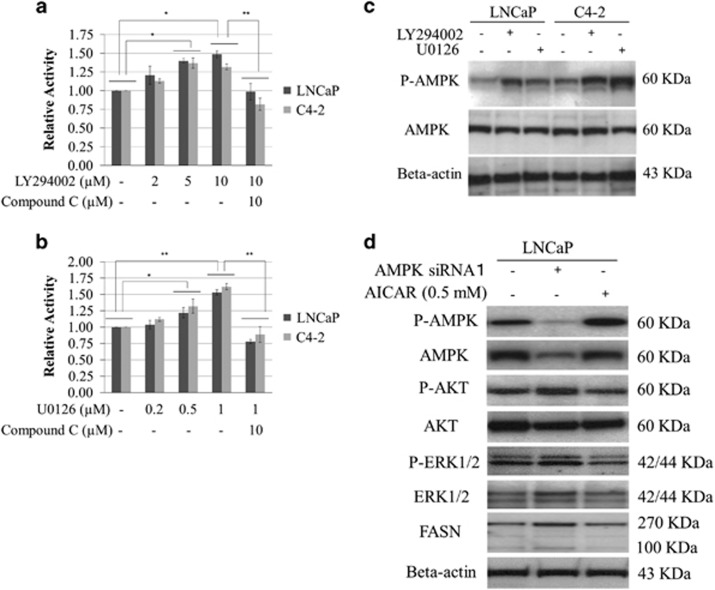
Crosstalk among PI3K, MAPK and AMPK signalling pathways related to FASN expression. (**a**, **b**) LNCaP and C4-2 cells were cultured with LY294002 and U0126 at the indicated concentration with or without 10 μM compound C for 1 h. The cells were washed and lysed, and the enzymatic activity of AMPK was measured using the CycLex AMPK Kinase Assay Kit. The enzymatic activity was assessed in comparison with that of the untreated cells. *P<0.05, **P<0.01. (**c**) LNCaP and C4-2 cells were cultured with 5 μM LY294002 and 0.5 μM U0126 for 1 h. Equal amounts of proteins from the cultured cells were subjected to anti-AMPK, anti-P-AMPK and anti-beta-actin. (**d**) LNCaP and C4-2 cells were cultured with 0.5 mM AICAR or treated with 50 nM AMPK siRNA for 1 and 6 h, respectively. Then, protein from the cultured cells was evaluated by western blot with anti-AMPK, anti-P-AMPK, anti-AKT, anti-P-AKT, anti-ERK, anti-P-ERK, anti-FASN and anti-beta-actin.

We next investigated the crosstalk among AKT, ERK and AMPK pathways for FASN expression in PCa cells. AMPK enzymatic activity was significantly increased in a dose-dependent manner in LNCaP and C4-2 cells treated with LY294002 or U0126, but the effects were abrogated in the presence of 10 μM compound C (Figures 3a and b). The finding was confirmed by western blotting (Figure 3c). Next, we examined the effect of either inhibiting or activating AMPK on the expression of AKT, ERK and FASN in PCa cells. FASN, P-AKT and P-ERK expression was upregulated in LNCaP cells by treatment with 50 nM of AMPK small interfering RNAs (siRNAs) for 6 h (Figure 3d, Supplementary Figure S4). When LNCaP cells were treated with 0.5 mM of AICAR for 1 h, expression of FASN, P-AKT and P-ERK was downregulated compared with the untreated condition or the condition under treatment with AMPK siRNA (Figure 3d).

### The role of FASN on cell proliferation and cell signalling in PCa cells

**Figure 4:**
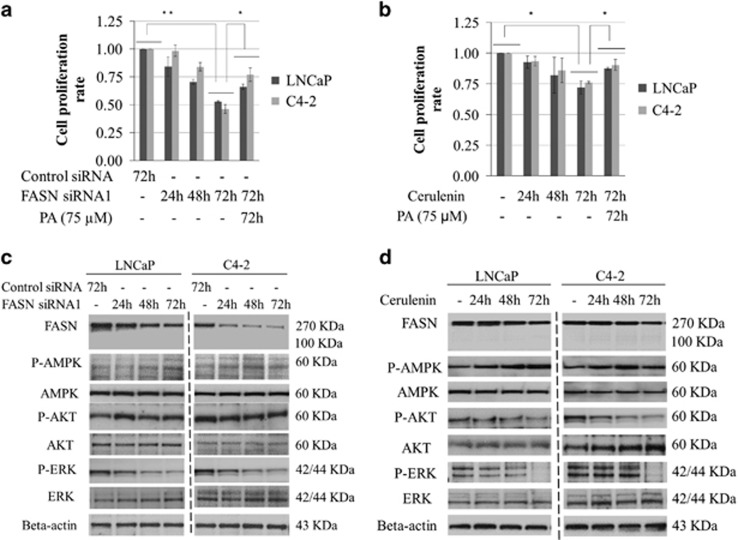
The role of FASN and related signalling pathways on PCa cell viability. LNCaP and C4-2 cells were cultured in a 96-well plate with Dulbecco's modified Eagle's medium (DMEM) containing 5% fetal bovine serum (FBS), and treated with 50 nM siRNA (**a**, **c**) and 10 μM cerulenin (**b**, **d**) with or without 75 μM PA for the indicated duration. The MTT assay was performed and cell viability was compared with that of cells treated with control siRNA or FBS (**a**, **b**). *P<0.05, **P<0.01. The cells were harvested, protein was extracted and an equal amount of each protein sample was subjected to western blotting using anti-FASN, anti-AKT, anti-P-AKT, anti-ERK, anti-P-ERK, anti-AMPK, anti-P-AMPK and anti-beta-actin (**c**, **d**).

Next, we investigated the role of FASN on PCa cell proliferation. Cell proliferation was significantly decreased in both LNCaP and C4-2 cells by treatment with both FASN siRNAs and cerulenin for 48 and 72 h, and the effects were partially rescued by adding 75 μM of palmitic acid (PA), the first fatty acid produced by FASN and the precursor to longer fatty acids (Figures 4a and b). In addition, we examined whether the inhibition of FASN affects AKT, ERK and AMPK activation in PCa cells. Western blotting revealed P-AKT and P-ERK protein expression to be downregulated, whereas P-AMPK expression was increased in the LNCaP and C4-2 cells after treatment with FASN siRNAs or cerulenin (Figures 4c and d; Supplementary Figure S5). These findings suggest that upregulated FASN increases tumour growth under HFD conditions. In addition, there may be a critical reciprocal stimulatory system in FASN expression and the PI3K and MAPK pathways associated with PCa progression. AMPK may have a suppressive role on the FASN/PI3K/MAPK signalling system.

### FASN expression was associated with adverse pathological findings in patients with PCa treated by radical prostatectomy

**Figure 5:**
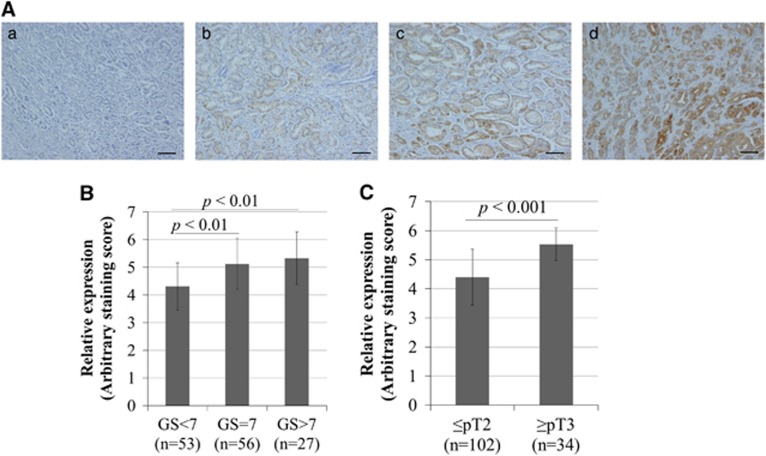
FASN expression was associated with the GS and pT stage in PCa patients. The slides of tissue sample of PCa from radical prostatectomy were immunohistologically stained using anti-human FASN-specific antibody, and the staining score calculated by scoring staining intensity and area. (**A**) These representative images show negative staining (a), low staining (b), moderate staining (c) and high staining (d). (**B**) The staining level of FASN was significantly higher in patients with high GS (GS=7, *n*=56; and GS >7, *n*=27) than in those with low GS (GS <7, *n*=53). *P*<0.01. (**C**) The FASN staining level in the surgical specimen was significantly higher in patients with pathological stage pT3 or higher than in those with ⩽pT2 (*P*<0.001).

To determine the clinical role of FASN on PCa progression, we performed FASN immunohistochemistry in patients with PCa treated by radical prostatectomy. Anti-FASN antibody showed that FASN was predominantly expressed in the cytoplasm of cancer epithelial cells. The staining level of FASN in PCa specimens was calculated according to the intensity and proportion of positive cells (Figure 5A). When the relationship between FASN expression and Gleason score (GS) was evaluated, the FASN staining level was significantly higher in PCa patients with a GS of 7 compared with a GS of ⩽6 (*P*<0.01; Figure 5B). Moreover, the staining level was significantly higher in PCa patients and a GS of ⩾8 than in those with a GS of ⩽6 (*P*<0.01; Figure 5B). The FASN staining level was significantly higher in patients with advanced pathological T (pT) stages (⩾pT3) of PCa than in those with more localized disease (⩽pT2) (*P*<0.001; Figure 5C).

### Downregulation of FASN in response to ADT and chemotherapy in the prostatectomy specimens of patients with PCa

**Figure 6:**
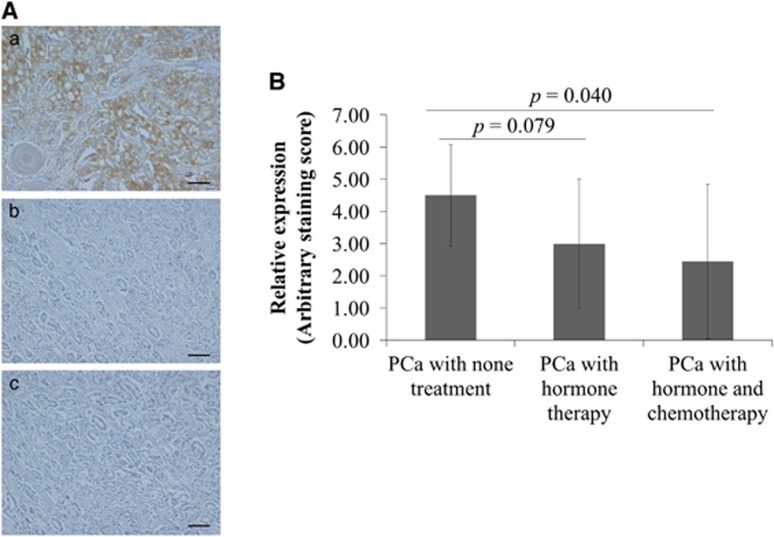
FASN expression decreased in response to ADT and chemotherapy in PCa patients. (**A**, **B**) The slides of tissue sample of another PCa from radical prostatectomy were stained with anti-human FASN-specific antibody, and the FASN staining level was scored. (**A**) Representative images of FASN expression. The three PCa patient types that underwent radical prostatectomy with or without neoadjuvant chemohormonal therapy were as follows: (a) no preoperative therapy, (b) neoadjuvant ADT and (c) neoadjuvant ADT with chemotherapy. (**B**) The FASN staining level was significantly lower in PCa subjected to neoadjuvant ADT or chemotherapy before radical prostatectomy when compared with untreated PCa.

To assess the changes in FASN expression in PCa patients treated with androgen deprivation therapy (ADT) and chemotherapy, we investigated the FASN staining level in radical prostatectomy specimens treated with or without neoadjuvant chemohormonal therapies. The patients enrolled in this study either (a) had not received any ADT or chemotherapy before prostatectomy (*n*=10), (b) had received ADT alone for 3–6 months (*n*=10) or (c) had received ADT for 3 months and docetaxel/estramustine phosphate chemohormonal therapy for 6 weeks (*n*=9) (Supplementary Figure S6). The FASN staining level tended to be lower in the PCa specimen when neoadjuvant therapy had been administered (*P=*0.079; Figures 6A and B). The FASN staining level was significantly lower in the sections from patients who had received neoadjuvant chemohormonal therapy than those without any preoperative treatment (*P=*0.040; Figures 6A and B).

## Discussion

In this study, the HFD enhanced tumour growth and FASN expression in the LNCaP xenograft mice, as expected. Interestingly, the serum FASN level was significantly lower in the HFD group than the LFD group and correlated inversely with tumour growth. Moreover, the HFD increased the levels of P-AKT, P-ERK and Ki67 positivity in xenograft tumours. Although this study provided no direct evidence to show that FASN activation is indispensable for HFD-induced enhancement of the LNCaP xenograft tumour growth, the higher level of P-AKT, P-ERK, Ki67 positivity and FASN in the HFD xenograft may support the results of *in vitro* experiments and consequently the significance of FASN. Through *in vitro* experiments, we also showed that FASN expression was decreased in the cytoplasm of PCa cells, whereas it was increased in the corresponding conditioned medium by treatment with PI3K and MAPK inhibitors (LY294002 and inhibitor U0126, respectively). These findings suggest that a HFD influences PCa progression through the upregulation of FASN expression and the inhibition of extracellular FASN release, which were also associated with upregulated PI3K and MAPK signalling. Alternatively, a HFD may activate PI3K and MAPK signalling and promote PCa cell growth through other mechanisms, in which case intracellular FASN was increased as the result of the enhanced PI3K and MAPK signalling. Either way, the results of our study suggest the presence of a critical stimulatory loop between FASN and PI3K and MAPK signalling.

In this study, intracellular FASN expression was higher and P-AMPK expression lower in xenograft tumours of the HFD group than the LFD group. In the *in vitro* studies, treatment with a pharmacological activator of AMPK (AICAR) resulted in decreased cellular expression levels of FASN and SREBP-1 and increased FASN in the conditioned medium in a dose-dependent manner. Moreover, both the enzymatic activity of AMPK and the protein expression of P-AMPK were significantly upregulated in PCa cells treated with LY294002 and U0126. In addition, the inhibition of AMPK by AMPK siRNA markedly stimulated P-ERK and P-AKT activation. Although no direct interaction between AMPK and PI3K or MAPK signalling was determined in this study, the results suggest the presence of a feedback system between AMPK and PI3K and MAPK signalling.

FASN has been shown to be upregulated in PCa, and its inhibition to be associated with decreased cell proliferation and increased apoptosis.^28^ Consistent with this, we found that PCa cell proliferation was decreased by treatment with FASN siRNA and a pharmacological inhibitor of FASN, cerulean. Moreover, this effect was partially rescued by PA, which is a major product mediated by FASN. Interestingly, the protein expression of P-AKT and P-ERK seemed to be downregulated, whereas P-AMPK was upregulated, by inhibiting FASN, whereas inhibition of either PI3K or MAPK was found to result in decreased intracellular FASN. This suggests that a stimulatory feedback loop may exist between FASN and PI3K/MAPK signalling, which may have a significant role in PCa progression. Equally, the AMPK system may work as a negative regulator to the stimulatory feedback loop of the FASN/MAPK/PI3K system. Furthermore, the stimulatory feedback loop may be closely linked to the PCa progression associated with HFD, although it remains to be clarified whether inhibiting the stimulatory feedback loop could prevent HFD-associated progression in PCa cells.

Ettinger *et al.*^29^ showed that FASN and SREBP-1, a transcriptional regulator of FASN, were dysregulated in the progression of castration-resistant PCa. As we demonstrated in the surgical specimens of human PCa, the FASN expression level was associated with both the GS and the pT stage. In addition, the FASN expression level was decreased in response to 3 months of hormone deprivation therapy or chemotherapy. This finding was consistent with the results reported by Ettinger *et al.*^29^, who found that SREBP-1 protein expression was decreased to the lowest level after hormonal therapy for 3 months, but that it increased thereafter. These findings strongly suggest that FASN may not only be a progressive marker but that it could also represent a critical therapeutic target in PCa.

Here, the extracellular secretion of FASN by PCa cells in the conditioned medium was enhanced by treatment with LY294002, U0126 and AICAR. Moreover, the serum FASN level was inversely correlated with tumour growth in LNCaP xenograft mice under HFD conditions. In addition, it was suggested that FASN was present in multiple forms in the conditioned medium, including intact and/or degraded forms. Although the exact biological role of extracellular FASN is unclear, studies have reported that FASN is present in the culture medium of breast cancer cells and in the serum of patients with breast cancer, and that extracellular FASN may therefore serve as a diagnostic and prognostic marker.^20, 21, 22^ FASN has seven catalytic domains, so FASN fragments probably have no activity because they lack essential domains.^30, 31^ Thus, intracellular rather than extracellular FASN may be a substantial modifier of metabolic alteration during PCa progression, whereas the extracellular FASN level may be an important diagnostic and prognostic marker.

In this study, we have demonstrated that a HFD increases both FASN and SREBP-1 expression and inhibits extracellular FASN release in the LNCaP xenograft mouse model. Furthermore, intracellular FASN was decreased and extracellular FASN release enhanced in the PCa cells by PI3K/MAPK inhibition or AMPK activation. FASN inhibition resulted in PI3K/MAPK downregulation and AMPK activation, whereas AMPK activation was associated with FASN downregulation and PI3K/MAPK inactivation. The results strongly indicate the presence of a critical regulatory feedback loop between the PI3K/MAPK system and AMPK signalling. Clinically, immunohistological evaluation of surgical specimens showed that high FASN expression was significantly associated with a high GS and an advanced pT stage of cancer. In addition, FASN expression was markedly decreased in the PCa response to ADT and chemotherapy. Therefore, FASN and its related kinetic pathways may be good targets for chemoprevention and therapy in PCa.

## Materials and methods

### Cell culture and reagents

Human PCa LNCaP cells were purchased from the American Type Culture Collection (Manassas, VA, USA) and the PCa C4-2 cells were kindly provided by Dr Leland WK Chung of Emery University.^32^ The cells were maintained in RPMI 1640 medium or Dulbecco's modified Eagle's medium (Invitrogen, Carlsbad, CA, USA) containing 10% fetal bovine serum and 1% penicillin–streptomycin. PI3K inhibitor (LY294002), MAPK inhibitor (U0126) and AMPK activator (AICAR) were purchased from Cell Signaling Technology (Boston, MA, USA). PA and FASN inhibitor (cerulenin) were purchased from Sigma (St Louis, MO, USA). The AMPK inhibitor compound C was purchased from EMD Millipore (Billerica, MA, USA).

### Animal study

The institutional review board of the Institutional Animal Care and Use Committee of Akita University Graduate School of Medicine approved all animal experiments procedures in this study. Six-week-old athymic BALB/c-nu/nu mice (*n*=24) were obtained from Japan SLC (Shizuoka, Japan) and fed an autoclaved CE-2 diet (Japan SLC); 1 × 10^6^ LNCaP cells were subcutaneously inoculated with ice-cold BD Matrigel (0.25 ml; BD Bioscience, Bedford, MA, USA) and RPMI medium (0.25 ml) in the hind limb. Four weeks after injection, mice with a palpable tumour were randomly assigned to either the HFD or the LFD group (*n*=12 per group). The HFD comprised 59.9% calories from fats, 21.4% from carbohydrates and 18.6% from proteins. The LFD comprised 9.5% calories from fats, 67.7% from carbohydrates and 22.8% from proteins (Supplementary Table S1). Body weight, tumour volume and food consumption were measured weekly, and tumour volume was calculated using the following formula:^5^ length (cm) × width (cm) × height (cm) × 0.5236. At week 14, mice were killed by CO_2_ asphyxiation, the xenograft tumours were excised and the mouse serum was separated.

### Serum analysis

The serum FASN concentration of xenograft mice was measured in duplicate using a sandwich enzyme-linked immunosorbent assay (ELISA) kit (Uscn Life Science Inc., Houston, TX, USA). The kit was a human-specific ELISA that does not cross-react with mouse FASN. Serum insulin was measured using an insulin enzyme immunoassay kit (Morinaga Institute of Biological Science, Tokyo, Japan), and serum IGF-1 was measured using a mouse/rat IGF-1 ELISA kit (R&D Systems, Minneapolis, MN, USA). Briefly, 100 μl of standards and the diluted serum samples were incubated in the human FASN, mouse insulin or mouse IgG-coated 96-well plates for 2 h. After washing, a 1-h incubation period was performed with a second biotinylated anti-human FASN, anti-mouse insulin or anti-mouse IGF-1 antibody and followed by a 30-min incubation period with streptavidin peroxidase (horseradish peroxidase). After washing to remove all unbound enzyme, colour was generated by adding tetramethylbenzidine and the reaction was stopped with a stop solution (2 N H_2_SO_4_). Concentrations of serum FASN, insulin and serum IGF-1 were calculated using standard curves.

### siRNA constructs

FASN siRNA1 (SI00059752), siRNA2 (SI0059759), siRNA3 (SI3082261) and luciferase siRNA (SI03650353) were purchased from Qiagen (Valencia, CA, USA), and AMPK siRNA1 and siRNA2 was purchased from Cell Signaling Technology. The luciferase siRNA was used as a control. Transfection of siRNAs was performed using Lipofectamine 2000 (Invitrogen). Cells were cultured in a 35-mm dish and treated with siRNA (50 nM) in reduced-serum Dulbecco's modified Eagle's medium. FASN and AMPK knockdowns were verified by western blotting.

### Quantitative reverse transcription–PCR

Total RNA was extracted from the cultured cells or the xenograft tumour tissues using the TRIzol reagent (Invitrogen). The following reverse transcription–PCR primers were used: *FASN*, forward 5′-CAG CCA TGG AGG AGG TGG TGA TT-3′, reverse 5′-CGA AGA AGG AGG CAT CAA ACC TA-3′ *SREBP-1*, forward 5′-ACG GCA GCC CCT GTA ACG ACC ACT GTG A-3′, reverse 5′-TGC CAA GAT GGT TCC GCC ACT CAC CAG G-3′ *AMPK* forward 5′-GAC AGC CGA GAA GCA GAA AC-3′, reverse 5′-AGG ATG CCT GAA AAG CTT GA-3′ and *beta-actin*, forward 5′-ATC TGG CAC CAC ACC TTC TA-3′, reverse 5′-CGT CAT ACT CCT GCT TGC TGA TCC ACA TCT GC-3′. The experiments were performed in triplicate.

### Cell proliferation assay

In total, 1 × 10^4^ cells were seeded in a 96-well plate and cultured in Dulbecco's modified Eagle's medium containing 5% fetal bovine serum without antibiotics. Then, the cells were treated with siRNA (50 nM) or cerulenin (10 μM) with or without PA (75 μM), and cultured for the indicated times. Cell proliferation was assessed using a nonradioactive 3-(4,5-dimethylthiazol-2-yl)-2,5-diphenyltetrazolium bromide (MTT)-based cell proliferation assay kit (Roche, Basel, Switzerland). Briefly, 10 μl of MTT (5 mg/ml: 1XPBS; component A) was added to each well and incubated for 4 h at 37 °C,Then, 100 μl of sodium dodecyl sulphate (component B) was added to each well and incubated for 24 h at 37 °C. Absorbance was measured at 570 nm using an ELISA reader (Bio-Rad, Tokyo, Japan). The experiments were performed in triplicate.

### Western blotting

Proteins were extracted from the cultured cells, supernatants and the xenograft tumours using Complete Lysis-M buffer (Roche). Equal amounts of protein were incubated with anti-FASN (BD Bioscience), anti-SREBP-1 (Santa Cruz Biotechnologies, Dallas, TX, USA), anti-AKT, anti-phospho-AKT (P-AKT, Ser^473^), anti-ERK1/2, anti-phospho-ERK1/2 (P-ERK1/2, Thr202/Tyr204), anti-AMPK, anti-phospho-AMPK (P-AMPK, Thr172) or anti-beta-actin (Cell Signaling Technology) antibody.

### Semiquantified estimation of AMPK activity

We seeded 2 × 10^5^ cells in a 35-mm dish and cultured them with LY294002 or U0126 in a dose-dependent manner for 1 h to maximum concentrations of 10 and 1 μM with or without 10 μM compound C, respectively. Then, the cells were washed and lysed, and the AMPK enzymatic activity in the lysates was measured by the CycLex AMPK Kinase Assay Kit (MBL, Nagoya, Japan). Briefly, 100 μl of cell lysates were incubated in the 96-well plates coated IRS-1-S789-peptide as AMPK substrate for 30 min at 30 °C. The wells were washed, and incubated with a anti-phospho-mouse IRS-1 S789 monoclonal antibody for 30 min, and followed with horseradish peroxidase-conjugated anti-mouse IgG at room temperature. After washing, colour was developed by adding tetramethylbenzidine and the reaction was stopped with a stop solution, and the absorbance was measured at 450 nm. The AMPK activity of each sample was measured and compared with that of the untreated control cells. The experiments were performed in triplicate.

### Immunohistochemistry

Slides containing tissue samples from 164 radical prostatectomy specimens were obtained from Akita University Hospital. The mean age, body mass index and preoperative prostate-specific antigen level of the patients with PCa were 66.4±4.5 years, 22.5±1.5 kg/m^2^ and 12.1±6.9 ng/ml, respectively. The Institutional Review Board and Ethics Committee of the Akita University Graduate School of Medicine approved all experiments in this study and we obtained written informed consent for the use of all human sample regarding this study project. The FASN mouse monoclonal antibody (Santa Cruz Biotechnologies) was used as the primary antibody at a dilution of 1:100. Immunohistochemical staining was performed as previously described.^33^ Evaluation and scoring were performed with the investigators (MH and HN) blinded to the patients' background and clinicopathological information. The FASN staining intensity in the cancer epithelium was scored on a semiquantitative scale, as follows: 0, negative; 1, low; 2, moderate; and 3, strong. The FASN staining area was also scored semiquantitatively, as follows: 0, negative (no staining); 1, low (<25%); 2, moderate (25–50%); and 3, high (>50%). The FASN staining score was determined by the FASN intensity and area scores. Sections of formalin-fixed paraffin-embedded xenograft tumours were stained with anti-FASN (1:100; BD Bioscience), anti-AKT (1:100; Cell Signaling Technology), anti-P-AKT (Ser^473^, 1:100; Cell Signaling Technology), anti-ERK1/2 (1:100; Cell Signaling Technology), anti-P-ERK1/2 (Thr202/Tyr204, 1:100; Cell Signaling Technology), anti-AMPK (1:100; Cell Signaling Technology) and anti-P-AMPK (Thr172, 1:100; Cell Signaling Technology) antibodies. After overnight incubation, the tissue sections were incubated with horseradish peroxidase-labelled anti-mouse or anti-rabbit antibody (1:5000). The staining intensity in the xenograft cancer cells was scored on a semiquantitative scale as follows: 0, negative; 1, low; 2, moderate and 3, strong. The xenograft tumour sections were probed with anti-Ki67 (1:800; Cell Signaling Technology), and the Ki67 expression levels were evaluated by counting of the Ki67-positive cells in 400–500 tumour cells in the area containing the highest density of Ki67-labelled tumour cells of the slides.

### Statistical analysis

Statistical analyses were performed using Microsoft Excel and SPSS version 12 (IBM Japan, Tokyo, Japan). All values are presented as mean±standard error. Differences between two groups in each experiment were evaluated using unpaired Student's *t-*tests. The Pearson correlation coefficient (*r*) was calculated to investigate the relationship between the serum FASN concentration and the tumour volumes of xenograft mice. A positive correlation was considered when *r* was >0.5. Differences were considered statistically significant if the *P*-value was <0.05.

## References

[bib1] Jemal A, Siegel R, Ward E, Murray T, Xu J, Thun MJ. Cancer statistics, 2007. CA 2007; 57: 43–66. 17237035 10.3322/canjclin.57.1.43

[bib2] Ito K. Prostate cancer in Asian men. Nat Rev Urol 2014; 11: 197–212. 24595118 10.1038/nrurol.2014.42

[bib3] Calle EE, Rodriguez C, Walker-Thurmond K, Thun MJ. Overweight, obesity, and mortality from cancer in a prospectively studied cohort of US adults. N Engl J Med 2003; 348: 1625–1638. 12711737 10.1056/NEJMoa021423

[bib4] Blair A, Fraumeni JF. Geographic patterns of prostate cancer in the United States. J Natl Cancer Inst 1978; 61: 1379–1384. 281545

[bib5] Narita S, Tsuchiya N, Saito M, Inoue T, Kumazawa T, Yuasa T et al. Candidate genes involved in enhanced growth of human prostate cancer under high fat feeding identified by microarray analysis. Prostate 2008; 68: 321–335. 18175332 10.1002/pros.20681

[bib6] Huang M, Narita S, Numakura K, Tsuruta H, Saito M, Inoue T et al. A high-fat diet enhances proliferation of prostate cancer cells and activates MCP-1/CCR2 signaling. Prostate 2012; 72: 1779–1788. 22514016 10.1002/pros.22531

[bib7] Venkateswaran V, Klotz LH. Diet and prostate cancer: mechanisms of action and implications for chemoprevention. Nat Rev Urol 2010; 7: 442–453. 20647991 10.1038/nrurol.2010.102

[bib8] Zadra G, Priolo C, Patnaik A, Loda M. New strategies in prostate cancer: targeting lipogenic pathways and the energy sensor AMPK. Clin Cancer Res 2010; 16: 3322–3328. 20423984 10.1158/1078-0432.CCR-09-1955 PMC3176306

[bib9] Tsukamoto Y, Wong H, Mattick JS, Wakil SJ. The architecture of the animal fatty acid synthetase complex. IV. Mapping of active centers and model for the mechanism of action. J Biol Chem 1983; 258: 15312–15322. 6654914

[bib10] Schweizer M, Roder K, Zhang L, Wolf SS. Transcription factors acting on the promoter of the rat fatty acid synthase gene. Biochem Soc Trans 2002; 30: 1070–1072. 12440974 10.1042/bst0301070

[bib11] Menendez JA, Lupu R. Fatty acid synthase and the lipogenic phenotype in cancer pathogenesis. Nat Rev Cancer 2007; 7: 763–777. 17882277 10.1038/nrc2222

[bib12] Swinnen JV, Esquenet M, Goossens K, Heyns W, Verhoeven G. Androgens stimulate fatty acid synthase in the human prostate cancer cell line LNCaP. Cancer Res 1997; 57: 1086–1090. 9067276

[bib13] Swinnen JV, Ulrix W, Heyns W, Verhoeven G. Coordinate regulation of lipogenic gene expression by androgens: evidence for a cascade mechanism involving sterol regulatory element binding proteins. Proc Natl Acad Sci USA 1997; 94: 12975–12980. 9371785 10.1073/pnas.94.24.12975 PMC24248

[bib14] Van de Sande T, De Schrijver E, Heyns W, Verhoeven G, Swinnen JV. Role of the phosphatidylinositol 3'-kinase/PTEN/Akt kinase pathway in the overexpression of fatty acid synthase in LNCaP prostate cancer cells. Cancer Res 2002; 62: 642–646. 11830512

[bib15] Baba Y, Nosho K, Shima K, Meyerhardt JA, Chan AT, Engelman JA et al. Prognostic significance of AMP-activated protein kinase expression and modifying effect of MAPK3/1 in colorectal cancer. Br J Cancer 2010; 103: 1025–1033. 20808308 10.1038/sj.bjc.6605846 PMC2965861

[bib16] Pandey V, Vijayakumar MV, Ajay AK, Malvi P, Bhat MK. Diet-induced obesity increases melanoma progression: involvement of Cav-1 and FASN. Int J Cancer 2012; 130: 497–508. 21387314 10.1002/ijc.26048

[bib17] Xiao R, Su Y, Simmen RC, Simmen FA. Dietary soy protein inhibits DNA damage and cell survival of colon epithelial cells through attenuated expression of fatty acid synthase. Am J Physiol Gastrointest Liver Physiol 2008; 294: G868–G876. 18239060 10.1152/ajpgi.00515.2007

[bib18] Moore RG, Lange TS, Robinson K, Kim KK, Uzun A, Horan TC et al. Efficacy of a non-hypercalcemic vitamin-D2 derived anti-cancer agent (MT19c) and inhibition of fatty acid synthesis in an ovarian cancer xenograft model. PLoS ONE 2012; 7: e34443. 22509304 10.1371/journal.pone.0034443 PMC3317945

[bib19] Puig T, Relat J, Marrero PF, Haro D, Brunet J, Colomer R. Green tea catechin inhibits fatty acid synthase without stimulating carnitine palmitoyltransferase-1 or inducing weight loss in experimental animals. Anticancer Res 2008; 28: 3671–3676. 19189648

[bib20] Wang YY, Kuhajda FP, Li J, Finch TT, Cheng P, Koh C et al. Fatty acid synthase as a tumor marker: its extracellular expression in human breast cancer. J Exp Ther Oncol 2004; 4: 101–110. 15500005

[bib21] Wang YY, Kuhajda FP, Cheng P, Chee WY, Li T, Helzlsouer KJ et al. A new model ELISA, based on two monoclonal antibodies, for quantification of fatty acid synthase. J Immunoassay Immunochem 2002; 23: 279–292. 12227415 10.1081/IAS-120013027

[bib22] Wang Y, Kuhajda FP, Li JN, Pizer ES, Han WF, Sokoll LJ et al. Fatty acid synthase (FAS) expression in human breast cancer cell culture supernatants and in breast cancer patients. Cancer Lett 2001; 167: 99–104. 11323104 10.1016/s0304-3835(01)00464-5

[bib23] Vazquez-Martin A, Fernandez-Real JM, Oliveras-Ferraros C, Navarrete JM, Martin-Castillo B, Del Barco S et al. Fatty acid synthase activity regulates HER2 extracellular domain shedding into the circulation of HER2-positive metastatic breast cancer patients. Int J Oncol 2009; 35: 1369–1376. 19885560 10.3892/ijo_00000455

[bib24] Fernandez-Real JM, Menendez JA, Moreno-Navarrete JM, Bluher M, Vazquez-Martin A, Vazquez MJ et al. Extracellular fatty acid synthase: a possible surrogate biomarker of insulin resistance. Diabetes 2010; 59: 1506–1511. 20299470 10.2337/db09-1756 PMC2874712

[bib25] Kuhajda FP. AMP-activated protein kinase and human cancer: cancer metabolism revisited. Int J Obes (Lond) 2008; 32(Suppl 4): S36–S41. 18719597 10.1038/ijo.2008.47 PMC7104469

[bib26] Oliveras-Ferraros C, Vazquez-Martin A, Fernandez-Real JM, Menendez JA. AMPK-sensed cellular energy state regulates the release of extracellular Fatty Acid Synthase. Biochem Biophys Res Commun 2009; 378: 488–493. 19032940 10.1016/j.bbrc.2008.11.067

[bib27] Tang FY, Pai MH, Chiang EP. Consumption of high-fat diet induces tumor progression and epithelial-mesenchymal transition of colorectal cancer in a mouse xenograft model. J Nutr Biochem 2012; 23: 1302–1313. 22221675 10.1016/j.jnutbio.2011.07.011

[bib28] Migita T, Ruiz S, Fornari A, Fiorentino M, Priolo C, Zadra G et al. Fatty acid synthase: a metabolic enzyme and candidate oncogene in prostate cancer. J Natl Cancer Inst 2009; 101: 519–532. 19318631 10.1093/jnci/djp030 PMC2664091

[bib29] Ettinger SL, Sobel R, Whitmore TG, Akbari M, Bradley DR, Gleave ME et al. Dysregulation of sterol response element-binding proteins and downstream effectors in prostate cancer during progression to androgen independence. Cancer Res 2004; 64: 2212–2221. 15026365 10.1158/0008-5472.can-2148-2

[bib30] Chakravarty B, Gu Z, Chirala SS, Wakil SJ, Quiocho FA. Human fatty acid synthase: structure and substrate selectivity of the thioesterase domain. Proc Natl Acad Sci USA 2004; 101: 15567–15572. 15507492 10.1073/pnas.0406901101 PMC524853

[bib31] Maier T, Leibundgut M, Ban N. The crystal structure of a mammalian fatty acid synthase. Science 2008; 321: 1315–1322. 18772430 10.1126/science.1161269

[bib32] Wu HC, Hsieh JT, Gleave ME, Brown NM, Pathak S, Chung LW. Derivation of androgen-independent human LNCaP prostatic cancer cell sublines: role of bone stromal cells. Int J Cancer 1994; 57: 406–412. 8169003 10.1002/ijc.2910570319

[bib33] Huang M, Narita S, Tsuchiya N, Ma Z, Numakura K, Obara T et al. Overexpression of Fn14 promotes androgen-independent prostate cancer progression through MMP-9 and correlates with poor treatment outcome. Carcinogenesis 2011; 32: 1589–1596. 21828059 10.1093/carcin/bgr182

