# Supplementary material for: Diet-induced alteration of fatty acid synthase in prostate cancer progression
Source: Oncogenesis. 2016 Feb 15;5(2):e195–. doi: 10.1038/oncsis.2015.42 (PMC5154344; doi:10.1038/oncsis.2015.42)
Supplement: Supplementary Information [file oncsis201542x1.doc]

**Diet-induced alteration of fatty acid synthase in prostate cancer progression**

Mingguo Huang^1, 4^, Atsushi Koizumi^1, 4^, Shintaro Narita^1, 4^, Takamitsu Inoue^1, 4^, Norihiko Tsuchiya^1^, Hiroki Nakanishi^2^, Kazuyuki Numakura^1^, Hiroshi Tsuruta^1^, Mitsuru Saito^1^, Shigeru Satoh^1^, Hiroshi Nanjo^3^, Takehiko Sasaki ^2, 4^ and Tomonori Habuchi*^1, 4^

**Supporting Information**

**Supplementary Table S1. Ingredients of Experimental Diets.**

**Supplementary Table S2. Food and caloric consumption of the LNCaP xenograft mice under HFD and LFD conditions.**

**Supplementary Figure S1. The tumour volume in the LNCaP xenograft mice under HFD and LFD conditions.**

LNCaP xenograft mice with palpable tumours were divided into HFD and LFD groups (12 mice/group). After the 14-week diet experiments, the tumour and serum samples were separated. The tumour volumes were measured, individually plotted for each animal and compared for the two diets. * *p* < 0.05

.

**Supplementary Figure S2. The expression of AKT, ERK and AMPK in the LNCaP xenograft mice under HFD or LFD conditions.**

Xenograft tumour sections from mice in the HFD and LFD groups underwent immunohistological staining with anti-human AKT, ERK and AMPK antibody (bar, 100 µm). (A and B) The immunohistostaining intensity in the xenograft cancer cells was scored on a semiquantitative scale and compared for the two diet groups.

**Supplementary Figure S3. The expression of Ki67 in the LNCaP xenograft under HFD or LFD conditions.**

(A) Xenograft tumour sections from mice in the HFD and LFD groups underwent immunohistological staining with anti-human Ki67 antibody (bar, 100 µm).

(B) The Ki67 positivity in the xenografts was significantly increased in the HFD group compared to LFD group (36.6 ± 3.6 and 27.8 ± 8.5 %, respectively). ***p <* 0.01.

**Supplementary Figure S4. The effects of AMPK siRNA1 and siRNA2 on FASN expression and AKT and ERK kinetic activity.**

The LNCaP cells were cultured in the 35-mm dish and treated with 50 nM of AMPK siRNA1 and siRNA2 or control siRNA for 24 h.

(A) An equal amount of protein from the cells was subjected with anti-human-AMPK, P-AMPK, AKT, P-AKT, ERK, P-ERK, and FASN antibodies.

(B) Total RNA was extracted from the cells and *AMPK* and *beta-actin* mRNA levels were measured by qRT-PCR, and the *beta-actin* mRNA level was used as the internal control.

**Supplementary Figure S5. The effects of FASN siRNA2 and siRNA3 on AKT, ERK and AMPK kinetic activity.**

The LNCaP cells were incubated in the 35-mm dish and treated with 50 nM of FASN siRNA2 and siRNA3 or control siRNA for 24 h.

(A) An equal amount of protein from the cells was subjected with anti-human-FASN, AMPK, P-AMPK, AKT, P-AKT, ERK, and P-ERK antibodies.

(B) Total RNA was extracted from the cells and *FASN* and *beta-actin* mRNA levels were measured by qRT-PCR, and the *beta-actin* mRNA level was used as the internal control.
