# Supplementary material for: Diet-induced alteration of fatty acid synthase in prostate cancer progression
Source: Oncogenesis. 2016 Feb 15;5(2):e195–. doi: 10.1038/oncsis.2015.42 (PMC5154344; doi:10.1038/oncsis.2015.42)
Supplement: Supplementary Figures [file oncsis201542x2.ppt]

## Slide 1
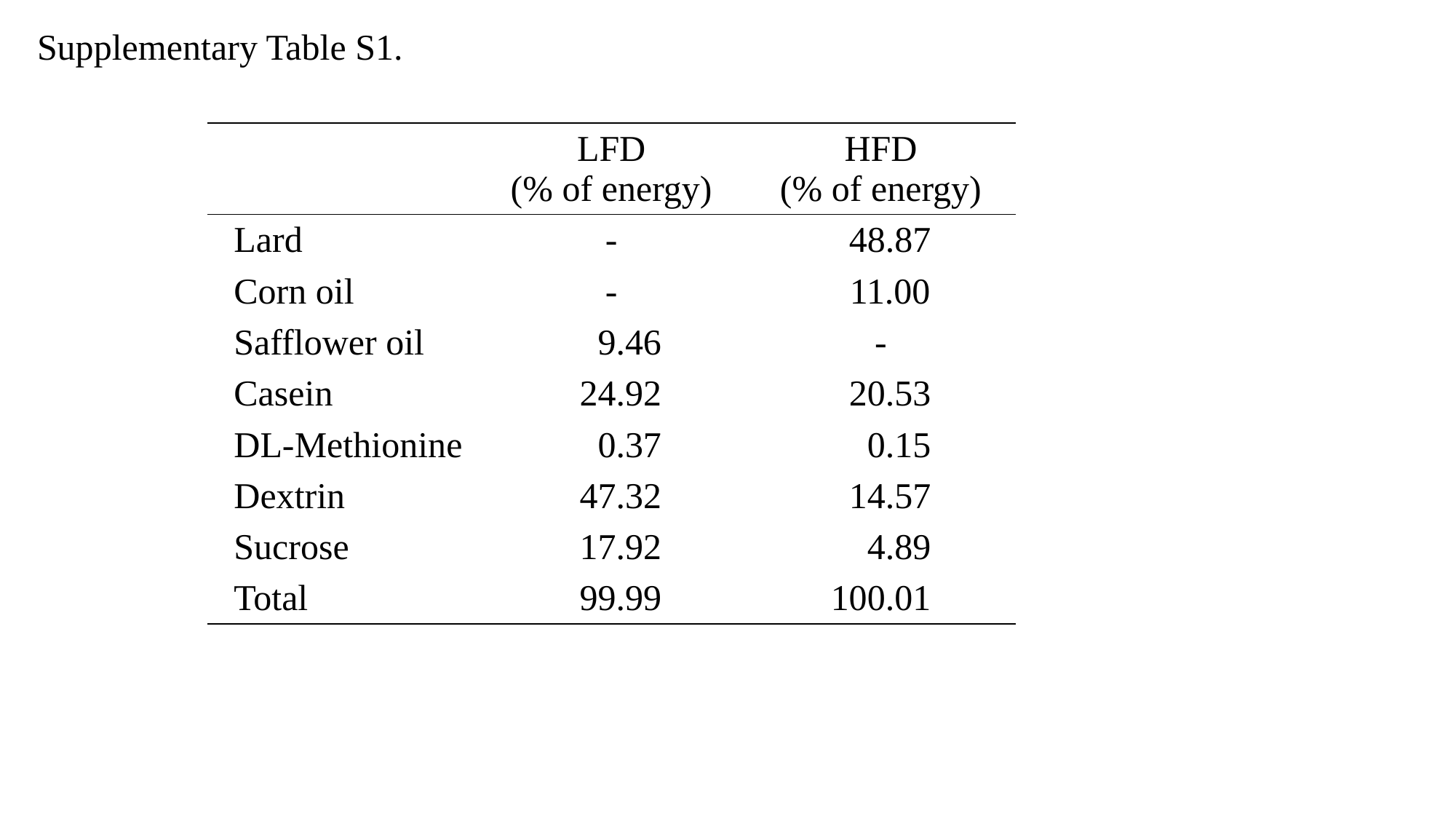

Supplementary Table S1.
| | LFD (% of energy) | HFD (% of energy) |
| --- | --- | --- |
| Lard | - | 48.87 |
| Corn oil | - | 11.00 |
| Safflower oil | 9.46 | - |
| Casein | 24.92 | 20.53 |
| DL-Methionine | 0.37 | 0.15 |
| Dextrin | 47.32 | 14.57 |
| Sucrose | 17.92 | 4.89 |
| Total | 99.99 | 100.01 |

## Slide 2
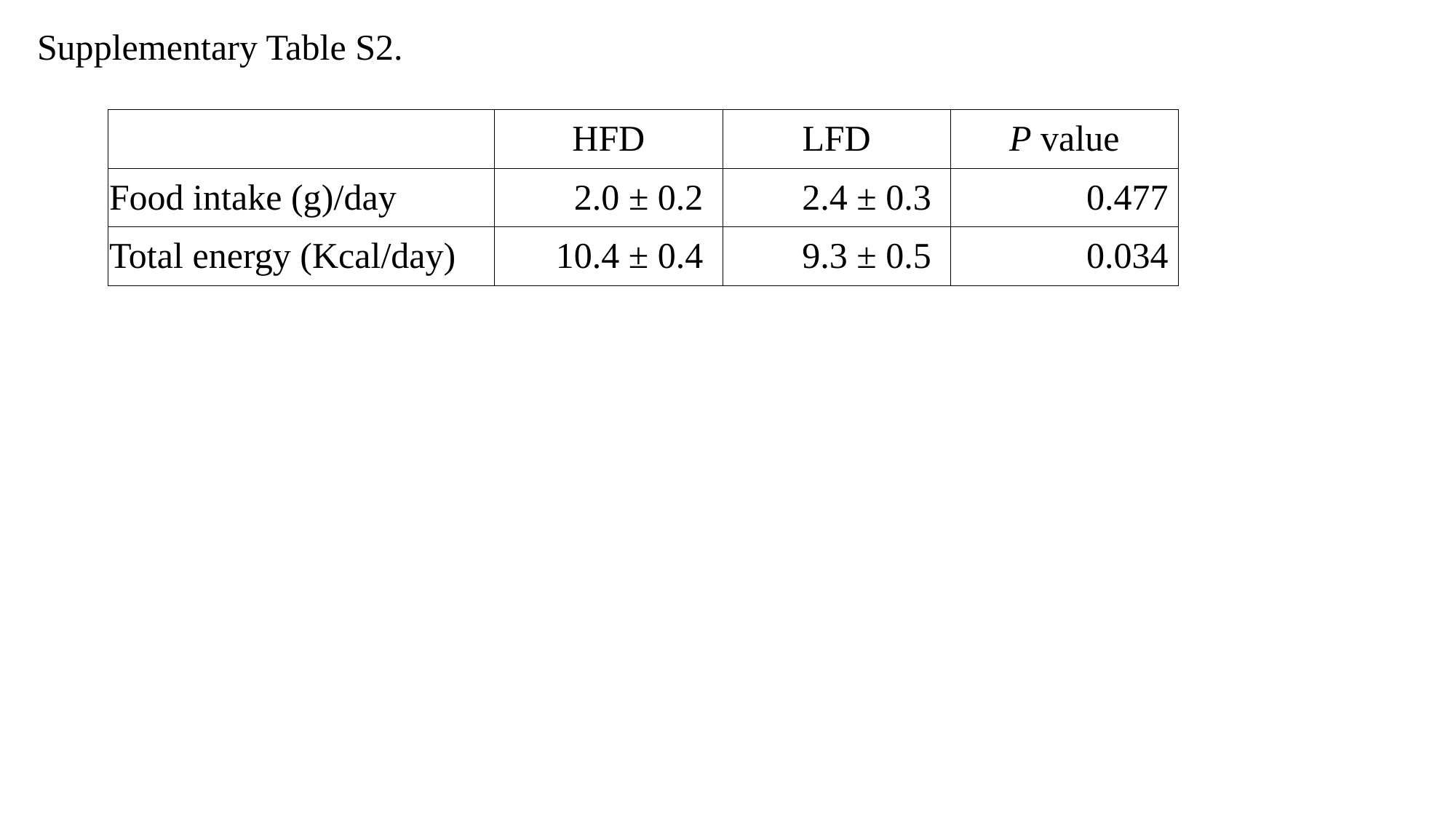

Supplementary Table S2.
| | HFD | LFD | P value |
| --- | --- | --- | --- |
| Food intake (g)/day | 2.0 ± 0.2 | 2.4 ± 0.3 | 0.477 |
| Total energy (Kcal/day) | 10.4 ± 0.4 | 9.3 ± 0.5 | 0.034 |

## Slide 3
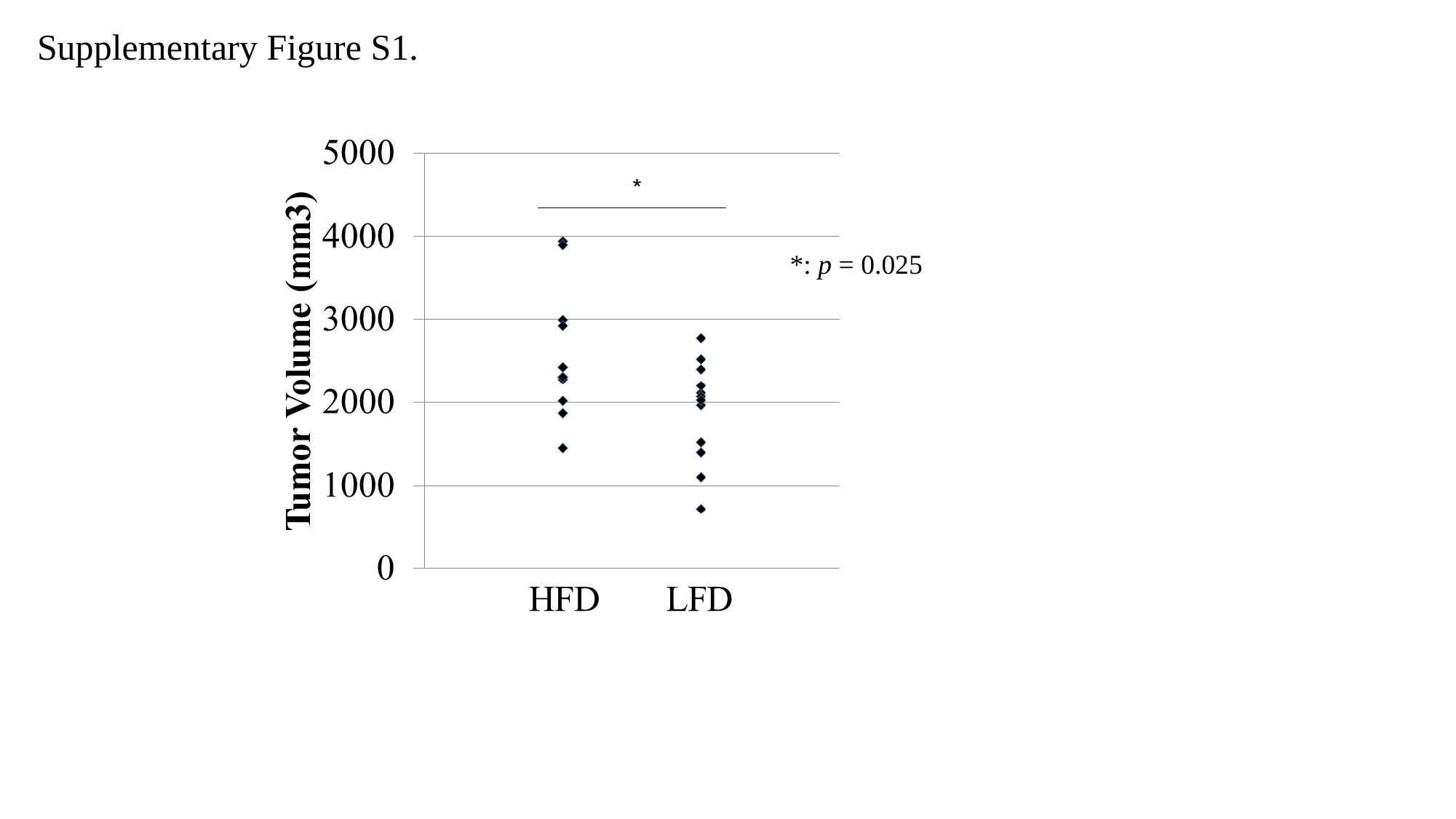

Supplementary Figure S1.
*: p = 0.025

## Slide 4
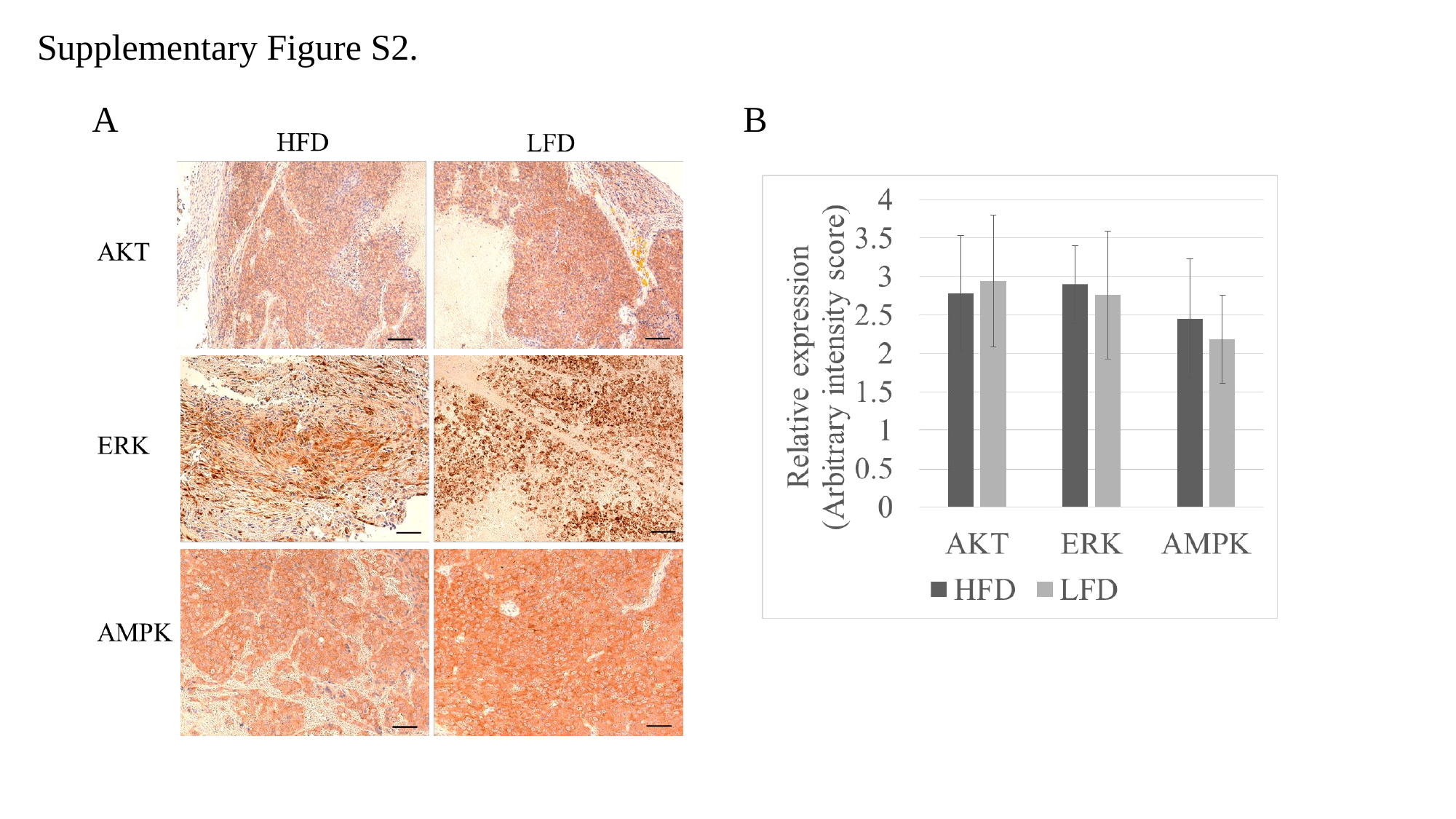

Supplementary Figure S2.
A
B

## Slide 5
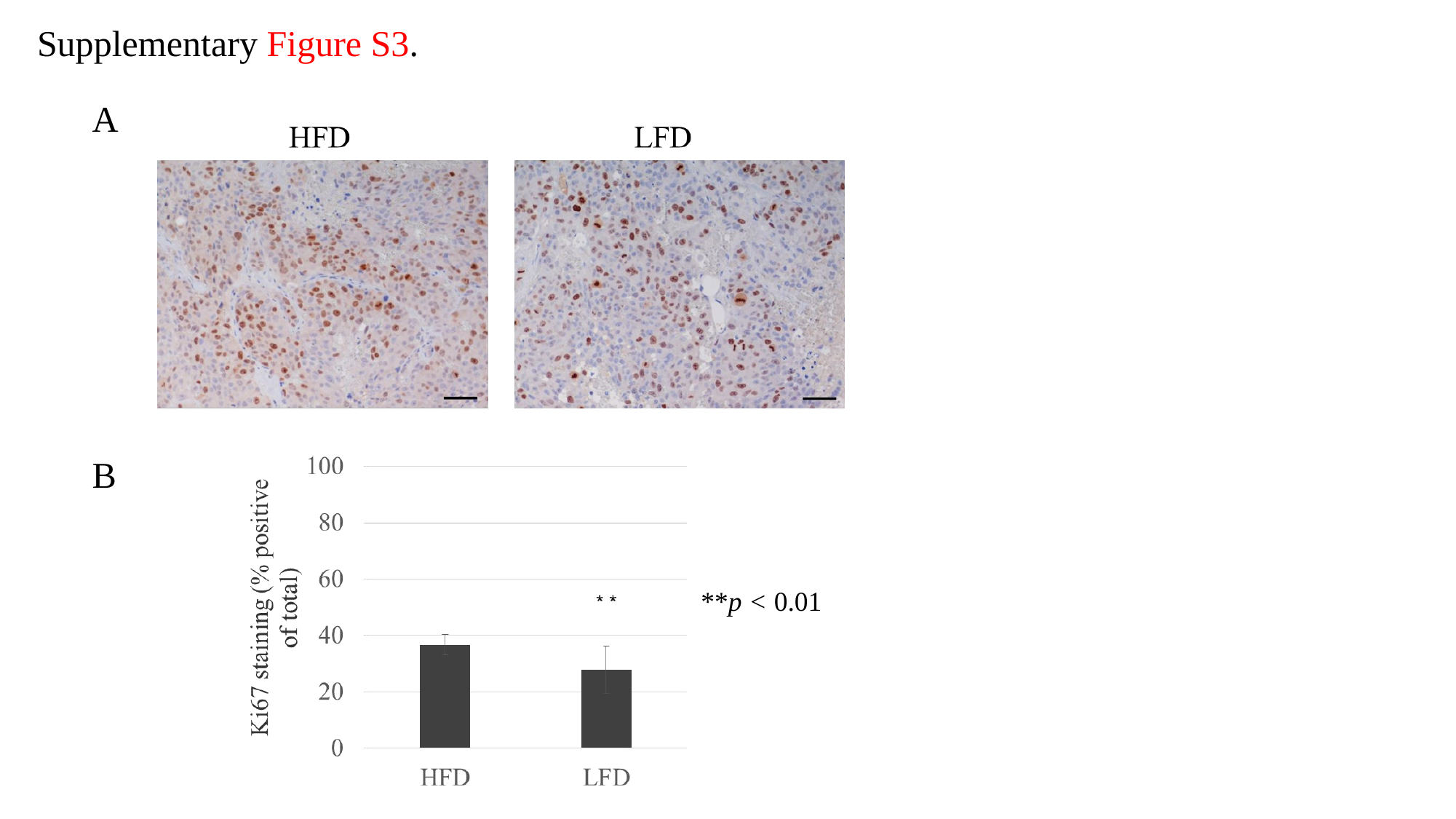

Supplementary Figure S3.
A
B
**p < 0.01

## Slide 6
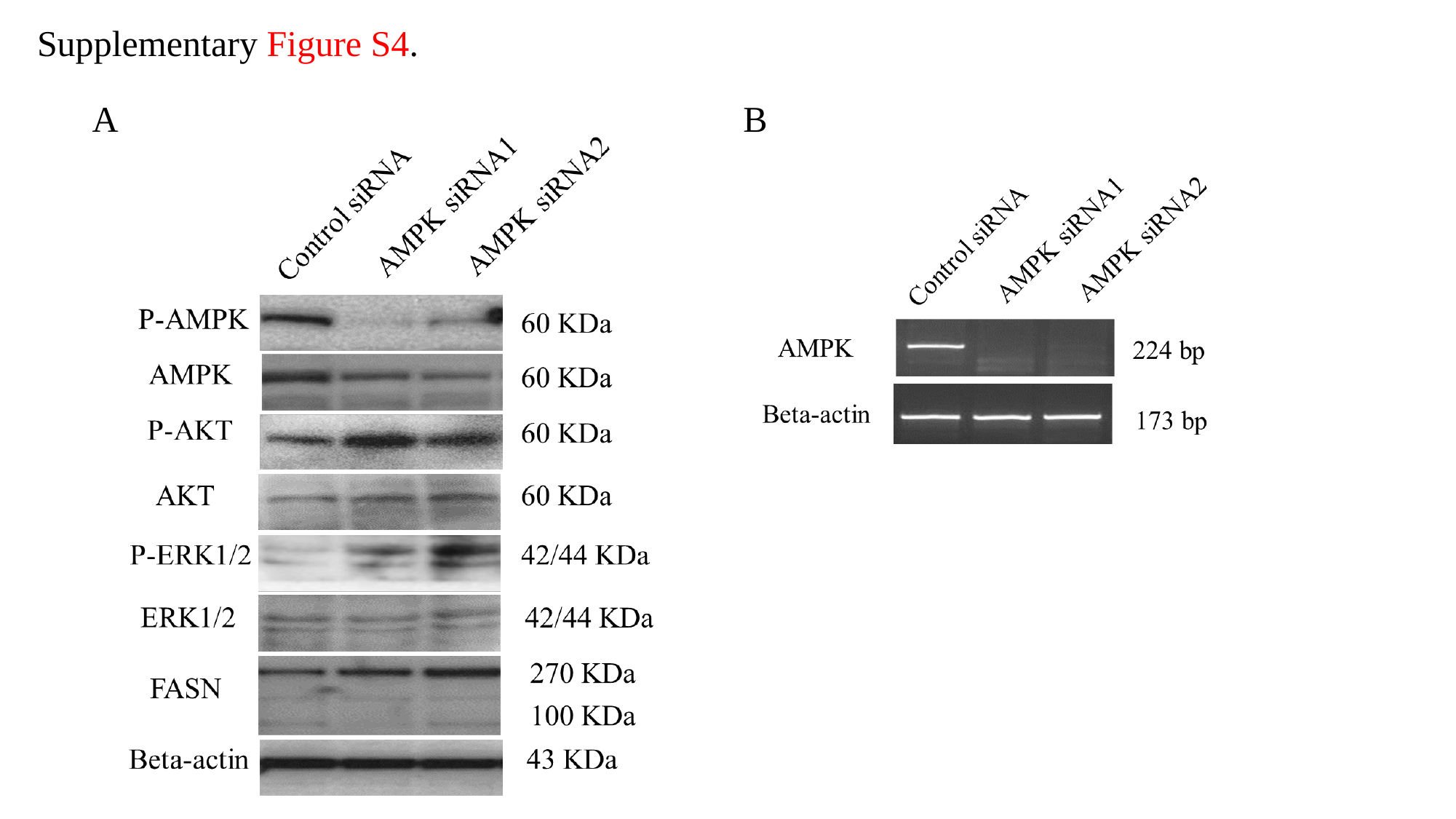

Supplementary Figure S4.
A
B

## Slide 7
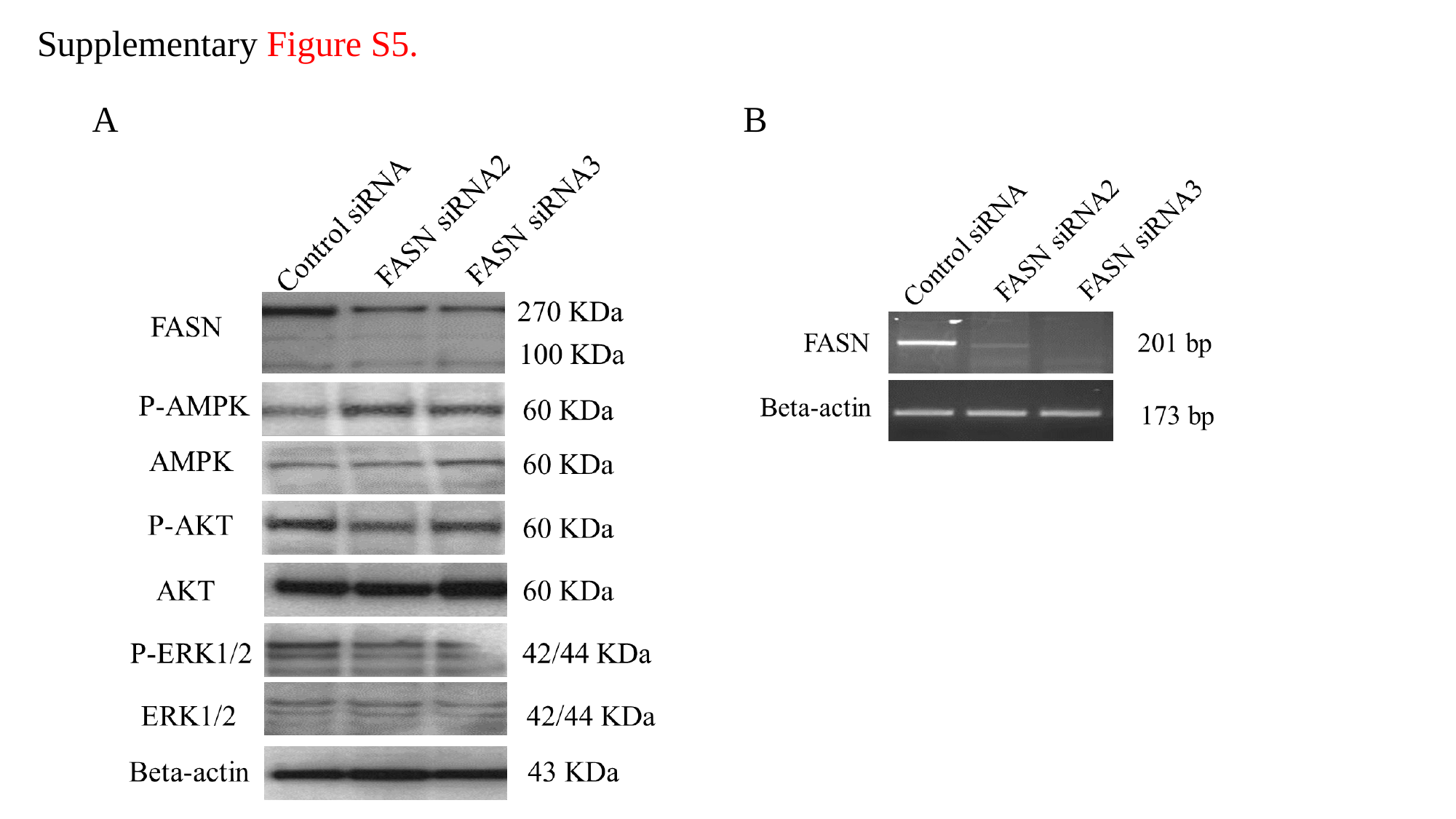

Supplementary Figure S5.
A
B
